# Fourier Transform Infrared Microspectroscopy Combined with Principal Component Analysis and Artificial Neural Networks for the Study of the Effect of β-Hydroxy-β-Methylbutyrate (HMB) Supplementation on Articular Cartilage

**DOI:** 10.3390/ijms22179189

**Published:** 2021-08-25

**Authors:** Izabela Świetlicka, Siemowit Muszyński, Carina Prein, Hauke Clausen-Schaumann, Attila Aszodi, Marcin B. Arciszewski, Tomasz Blicharski, Mariusz Gagoś, Michał Świetlicki, Piotr Dobrowolski, Katarzyna Kras, Ewa Tomaszewska, Marta Arczewska

**Affiliations:** 1 Department of Biophysics, Faculty of Environmental Biology, University of Life Sciences in Lublin, 20-950 Lublin, Poland; siemowit.muszynski@up.lublin.pl; 2 Center for Applied Tissue Engineering and Regenerative Medicine-CANTER, Munich University of Applied Sciences, 80335 Munich, Germany; carina.prein@uwo.ca (C.P.); hauke.clausen-schaumann@hm.edu (H.C.-S.); 3 Laboratory of Cartilage Development, Diseases and Regeneration, Department for Orthopaedics and Trauma Surgery, Musculoskeletal University Centre Munich (MUM), University Hospital, LMU Munich, 82152 Planegg, Germany; attila.aszodi@med.uni-muenchen.de; 4 Center for Nanoscience-CeNS, 80799 Munich, Germany; 5 Department of Animal Anatomy and Histology, University of Life Sciences in Lublin, 20-950 Lublin, Poland; mb.arciszewski@wp.pl (M.B.A.); katarzyna.kras@up.lublin.pl (K.K.); 6 Chair and Department of Rehabilitation and Orthopedics, Medical University in Lublin, 20-090 Lublin, Poland; tomasz.blicharski@umlub.pl; 7 Department of Cell Biology, Maria Curie Sklodowska University, 20-031 Lublin, Poland; mariusz.gagos@poczta.umcs.lublin.pl; 8 Department of Biochemistry and Molecular Biology, Medical University of Lublin, 20-093 Lublin, Poland; 9 Department of Applied Physics, Faculty of Mechanical Engineering, Lublin University of Technology, 20-618 Lublin, Poland; m.swietlicki@pollub.pl; 10 Department of Functional Anatomy and Cytobiology, Faculty of Biology and Biotechnology, Maria Curie-Sklodowska University, 20-033 Lublin, Poland; piotr.dobrowolski@poczta.umcs.lublin.pl; 11 Department of Animal Physiology, Faculty of Veterinary Medicine, University of Life Sciences in Lublin, 20-950 Lublin, Poland; ewaRST@interia.pl

**Keywords:** FTIR microspectroscopy, atomic force microscopy, β-hydroxy-β-methylbutyrate supplementation, animal model, articular cartilage, collagen, proteoglycans, principal component analysis, artificial neural networks

## Abstract

The potential of Fourier Transform infrared microspectroscopy (FTIR microspectroscopy) and multivariate analyses were applied for the classification of the frequency ranges responsible for the distribution changes of the main components of articular cartilage (AC) that occur during dietary β-hydroxy-β-methyl butyrate (HMB) supplementation. The FTIR imaging analysis of histological AC sections originating from 35-day old male piglets showed the change in the collagen and proteoglycan contents of the HMB-supplemented group compared to the control. The relative amount of collagen content in the superficial zone increased by more than 23% and in the middle zone by about 17%, while no changes in the deep zone were observed compared to the control group. Considering proteoglycans content, a significant increase was registered in the middle and deep zones, respectively; 62% and 52% compared to the control. AFM nanoindentation measurements collected from animals administered with HMB displayed an increase in AC tissue stiffness by detecting a higher value of Young’s modulus in all investigated AC zones. We demonstrated that principal component analysis and artificial neural networks could be trained with spectral information to distinguish AC histological sections and the group under study accurately. This work may support the use and effectiveness of FTIR imaging combined with multivariate analyses as a quantitative alternative to traditional collagenous tissue-related histology.

## 1. Introduction

Articular cartilage (AC) is a non-vascular type of connective tissue covering the end of bones forming synovial joints to facilitate the distribution of loads across articular surfaces with a low frictional coefficient and buffer vibration [1]. The extracellular matrix (ECM) of AC is composed of a dense network of fibrillary collagen (primarily, type II collagen) that is associated with other non-collagenous proteins and proteoglycans (PGs) [2,3]. Structurally, the mature, non-calcified AC tissue demonstrates zonal heterogeneity due to the orientation of the collagen fibrils and molecular composition, thus it is subdivided into three histological zones (superficial zone (SZ), middle zone (MZ) and deep zone (DZ), of which the latter is separated from the calcified AC zone by the tidemark) [4]. In the relatively thin SZ with the lowest PGs content [5] but a high level of collagen, collagen fibrils are oriented in parallel to the AC surface. Such an arrangement is responsible for most of the tensile stiffness of cartilage. In the MZ (ca. 60% of total AC thickness), collagen fibrils are mostly randomly organized, while in the DZ they are aligned perpendicular to the AC surface [5,6]. The specific structural framework of the collagen fibril network, their organization and the alterations in the content and spatial distribution of the main molecular components are responsible for the tensile biomechanical properties of AC [7,8]. The concentration of PGs is unevenly distributed throughout the tissue depth and reaches its maximum in the DZ. Among the PG family, largely aggregating aggrecan is considered the most crucial to the proper functioning of AC. It contains covalently attached side chains of the glycosaminoglycans (GAGs), chondroitin sulphate (CS) and keratin sulphate (KS) linked to a protein core. The CS chains of aggrecan play a major role in absorbing free ions and water into cartilaginous tissues, which provides the basis of its hydrodynamic viscoelastic properties as a weight-bearing tissue [9]. In this way, PGs are mainly responsible for the resiliency and compressive properties of AC. Unfortunately, AC, if damaged, displays a limited ability to self-regenerate [10,11]. Therefore, there is an ongoing intensive search for novel therapies, including preventive interventions and therapeutic solutions that can stimulate tissue regeneration and reduce cartilage degeneration.

HMB (β-hydroxy-β-methylbutyrate) is a natural, bioactive metabolite of the essential amino acid leucine. It has recently gained popularity as a dietary supplement due to its many beneficial effects on the skeletal system of animals via protective, anti-catabolic mechanisms and a direct influence on protein synthesis [12,13,14]. HMB has been shown to reduce protein breakdown by decreasing proteasome expression and proteasome enzyme activity, inhibit caspase up-regulation and limit apoptosis of muscle nuclei [15,16]. Moreover, Santos-Fandila et al. [17] reported in the brain microdialysis experiment that after being orally administered, HMB is able to cross the blood-brain barrier, which opens up new ways for its nutritional interventions, particularly concerning anti-ageing cognitive benefits. Regarding AC tissue, several studies have shown that the supplementation of HMB affects the distribution and structure of cartilage components [18,19,20,21]. The more significant changes after prenatal and maternal HMB-treatment of animals were seen in enhancing proteoglycan content and an intensive process of collagen synthesis. 

The fundamental parameters such as collagen content, its integrity, the orientation of collagen fibers and PGs content provide information about the AC quality [3]. However, the spatial distribution of collagen and PGs is hardly measurable with conventional biochemical methods. To address this problem, techniques that can visualize the spatial distribution of AC molecular components are required. Fourier transform infrared microspectroscopy, being a powerful technique, can quantitatively evaluate the distribution of chemical composition of biological tissues. It gives the possibility of simultaneously achieving the collection of infrared spectra and spectral maps based on the absorbance of a specific molecular species with satisfactory spatial and spectral resolutions [22]. So far, FTIR spectroscopy and its combination with microscopic visualization of the AC tissue have been successfully applied both to evaluate the relative changes in its composition and the spatial distribution of main AC components [23,24,25,26]. The development of FTIR spectroscopy in the AC research to provide a new strategy for rapid diagnosis of degenerative disease was reported elsewhere [27,28,29,30] and demonstrated by the changes in the spatial distribution of various components of AC mapped by FTIR microspectroscopy [31]. Using advanced multivariate data analysis methods, FTIR microspectroscopy can reveal hidden structures within spectral data concerning the tissue composition and structure, thereby helping improve medical diagnostics of cartilage diseases or monitor therapeutic progress [31]. Yin et al. [32] applied FTIR imaging to examine the collagen and PG levels and their depth dependence on healthy and osteoarthritis-affected cartilages. This technique has also been used to assess chemical changes associated with load, disuse, degradation and repair in cartilage and micro-damage in bone [33,34].

The present study aimed to involve multiple techniques, including FTIR microspectroscopy and atomic force microscopy (AFM), and combine them with multivariate methods such as principal component analysis (PCA) and artificial neural networks (ANNs) to reveal the variations in the structure and properties of cartilage tissue. We developed a swine model to investigate in vivo the influence of HMB supplementation during the fetal period on articular cartilage. The pig provides an attractive model to human health research priorities and nutritional studies because of many similarities to humans in structure and function, including the size of internal organs, feeding patterns, drug metabolism and dietary habits [35]. There is a general acceptance of the pig as a biomedical model after sequencing the complete swine genome and finding a high sequence homology to humans (ca. 60%) [35,36]. To our knowledge, there is no investigation regarding the HMB-related spatial distribution of collagen and proteoglycans in the extracellular matrix of cartilage tissue utilizing FTIR imaging and methods of multivariate data analysis. 

## 2. Results and Discussion

Articular cartilage is recognized as a tissue with structure, composition and functional properties that relevantly change during skeletal maturation. Observed alterations include but are not limited to increasing collagen network density, reorganization of chondrocytes and decrease in cellularity [37]. β-hydroxy β-methylbutyrate, administered during the prenatal period, was proved to enhance the mechanical, morphological and physiological features of tissues of offspring [20,38,39,40,41,42]. Since the mechanism of HMB action remains unknown, it is hypothesized that HMB could affect protein metabolism acting on several levels, e.g., inhibiting proteolysis, attenuating depression in protein synthesis, metabolic inhibitors and promoting protein synthesis [43,44,45,46], which is manifested by modifications in skeletal muscle structure as well as by changes in the mechanical and morphological properties of bone [18,20,40,42,47] and teeth [41]. Therefore, the first step of our research was to apply FTIR microspectroscopy to map the changes in the spatial distribution of AC components. 

**Figure 1:**
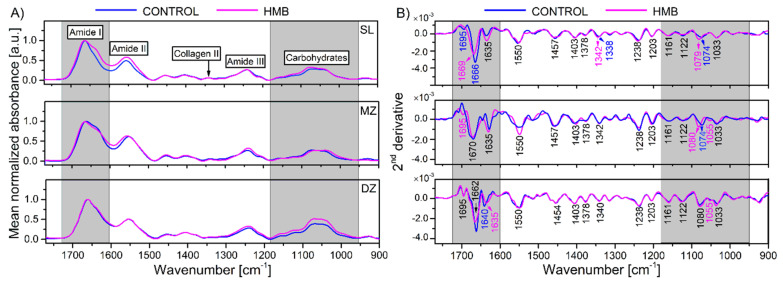
(**A**) Mean (*n* = 75 spectra) FTIR spectra of the cartilage matrix from control (blue line) and HMB-treated pigs (magenta line) in three regions after background correction and normalization based on the amide I band intensity, and (**B**) mean second-derivative spectra from the same samples calculated using the Savitzky-Golay algorithm with nine smoothing points. The second-derivative band assignments were given in Table 1. The band positions in black indicate the same values for all samples. HMB—supplemented group, SZ—superficial zone, MZ—middle zone, DZ—deep zone.

**Figure 2:**
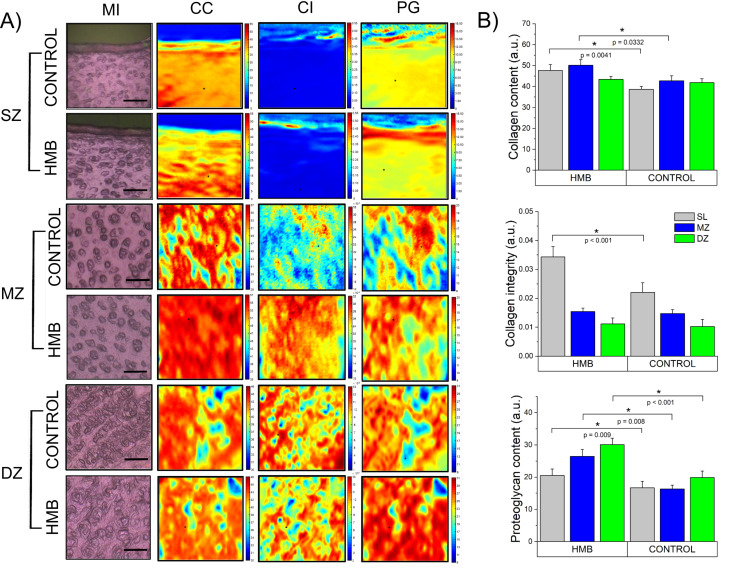
(**A**) The visible light microscopic images (MI) and representative chemical maps of porcine cartilage in three histological zones: superficial zone (SZ), middle zone (MZ) and deep zone (DZ), along with (**B**) the histograms of structural parameters characterizing articular cartilage: collagen content, collagen integrity and proteoglycans content with statistically significant differences between groups (at *p* < 0.05) marked with *. Chemical maps were constructed by integrating the area under the amide I band 1700−1600 cm^−1^ (CC) as the ratio of the area of the CH_2_ side chain of collagen 1360–1325 cm^−1^ absorption to the amide II 1590–1490 cm^−1^ (CI) and by integrating the area of the region assigned to the proteoglycans sugar ring 1140–965 cm^−1^ (PG) in control and HMB-treated samples. The color scale indicates the pixel values for all parameters. These maps were typical of all samples. The scale bars correspond to 20 μm. HMB—supplemented group, SZ—superficial zone, MZ—middle zone, DZ—deep zone, CC—collagen content, CI—collagen integrity, PG—proteoglycans content.

Figure 1A shows the average infrared absorption spectra collected from the superficial, middle and deep zones of cartilage, corresponding to the areas shown in Figure 2A. The characteristic bands of collagen and PGs cover the range of 1800–960 cm^−1^, including the amide I (1700–1600 cm^−1^), the amide II (1600–1500 cm^−1^), the amide III (1300–1200 cm^−1^) and the C–O stretching vibrations of the carbohydrate residues in collagens and PGs (1140–960 cm^−1^) [48]. The bands located between the spectral range of 1500–1300 cm^−1^ are mixed and relate to CH_2_ and CH_3_ vibrations and CH_2_ side chains vibrations of collagen [32,33]. More importantly, the bands located at 1338 cm^−1^ and in the region of 1080–1060 cm^−1^ are the most suitable for distinguishing collagen and PGs [49]. Since the absorption bands in the spectra of biological samples often overlap each other and the bands originating from collagen and other molecules of AC, such as chondroitin sulphate, are located in the same region, the second derivative method has been applied to enhance resolution in identifying subbands and better evaluate the spectral changes [50,51]. Rieppo et al. [29] reported a practical approach based on a second derivative analysis of collagen and PGs for the analysis of articular cartilage composition. The most significant peaks obtained from the second derivative spectra are shown in Figure 1B, while the assignments of the minima found in the 2nd derivative spectra are presented in Table 1.

Significant differences in the mean second-derivative spectra were found in the amide I band of the collagen helix and at the carbohydrates regions commonly used to estimate collagen and proteoglycan contents. The bands localized at 1695 cm^−1^, 1635 cm^−1^ and about 1660 cm^−1^ were assigned as characteristics for β-sheet and α-helix structures of amide I. The increase in the band intensity at 1635 cm^−1^ may indicate the presence of proteins with a more β-sheet structure for the HMB-supplemented samples in the SZ compared to the control. Moreover, a more prominent intensity of this band was observed in collagen-rich tissues due to the cross-linked collagen fibers [52]. In the mouse model, the presence of a 1635 cm^−1^ band indicated proteoglycan synthesis by surface activated chondrocytes [53]. Hence, the more reliable explanation of these alterations could cover newly synthesized collagen produced by activated chondrocytes or other unknown cartilage proteins observed in the HMB-supplemented samples, especially in the superficial zone. The obtained results are consistent with our previous work, in which significantly changed collagen structure in the articular cartilage of maternal HMB-supplemented newborn piglets was reported [18]. On the other hand, a higher relative contribution of extended β-sheets was observed in the dried tissue matrix [54]. It is worth mentioning that some limitations due to air drying of AC samples before FTIR analysis should be taken into account, especially in evaluating the amide I band. Water plays an essential role in maintaining the extracellular matrix, and dehydration may cause some changes in the structural organization of molecular components constituting articular cartilage, mainly collagen [55]. Kemp et al. reported that collagen fibril D-spacing decreases with dehydration, contributing to compromised mechanical integrity [56]. In the FTIR analysis of fully hydrated tissues, the absorbance band from water’s bending vibration near 1640 cm^−1^ overlaps seriously with the amide I band; therefore, tissue samples are generally dehydrated before analyses [31,48,57,58].

No significant differences in band locations were found in the region between 1500 cm^−1^ and 1200 cm^−1^, except for the spectral shift (~4 cm^−1^) of a band at 1338 cm^−1^ assigned to the CH_2_ side-chain vibrations in the case of HMB-supplemented samples. This band is commonly used to qualitatively estimate the integrity of the collagen network because it is not present in the pure PG spectrum [49,57]. The absorption intensity of the PG-related second derivative bands increased, especially for the DZ zone. In addition, a band at 1074 cm^−1^ showed the variability with the larger spectral shift towards higher wavenumbers in the HMB supplemented groups compared to the control. Interestingly, a band at 1055 cm^−1^ presented only in the DZ zone of the control group, while in the case of HMB-supplemented samples, it was observed in low depth regions of cartilage.

As shown in Figure 2A, chemical maps present cartilage samples as distributions of functional groups characteristic of the selected components of cartilage, namely collagen and proteoglycans. Chemical maps converted into quantities proportional to the integrated area of the chosen bands were used to characterize its structural parameters quantitatively. Mean values of collagen content (CC), collagen integrity (CI) and proteoglycans content (PG) were compared between the control and HMB groups in each zone (complete data available in the Supplementary Material, Figures S1–S3).

Obtained results show (Figure 2B) that in the group supplemented with HMB during the prenatal period, the collagen content increased in the SZ by more than 23% and in the MZ by about 17%, while no changes in the deep zone compared to the control group were observed. In the experimental group, a significant (approximately 55%) increase in the integrity of collagen in the surface zone were also detected. Interestingly, all three zones proved to be influenced by HMB administration when proteoglycans content was considered. A significant increase was observed in all of the examined areas; however, the highest ones were registered in the middle (62% compared to the control) and deep (52% compared to the control) zones. Achieved results are in line with histological proteoglycan staining (Supplementary Material, Figure S4).

It is proven that any modification in tissue organization is reflected in changes in mechanical features such as elasticity, stiffness and others [37]. Furthermore, mechanical properties measured in the nanoscale are recognized to be highly correlated with the structure of biological materials determined with spectral methods [60]. To examine whether structural alterations influenced tissue elasticity, AFM nanoindentation measurements were carried out.

**Figure 3:**
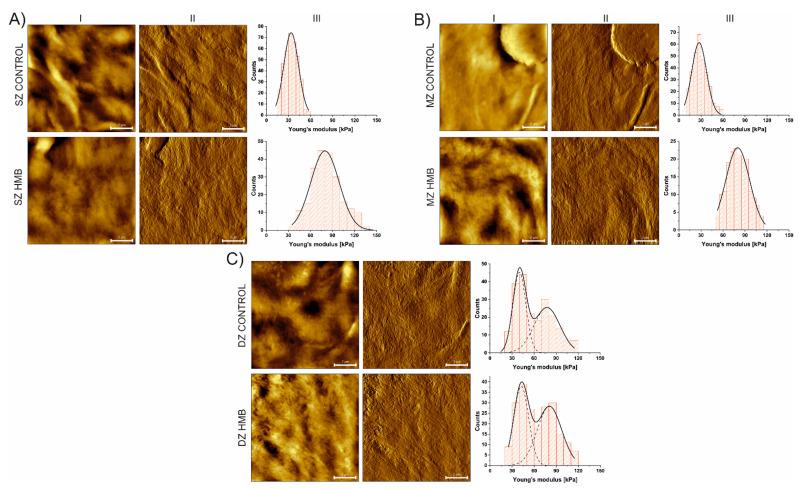
Height (I) and vertical deflection (II) images of AC zones with corresponding histograms of Young’s modulus distribution (III) determined for SZ (panel **A**), MZ (panel **B**) and DZ (panel **C**) with Gaussian functions fitted. Scale bars are equal to 2 µm. HMB—supplemented group, SZ—superficial zone, MZ—middle zone, DZ—deep zone.

Conducted AFM analysis revealed differences in the mechanical properties of articular cartilage collected from animals administered with HMB during the prenatal period when compared to the control. A shift towards higher values of Young’s modulus was detected for all the zones (Figure 3, Table 2), with the greatest being observed for the superficial and middle zones. According to our previous work [19], where the higher presence of collagen fibers (indicating higher collagen maturity), increased AC thickness and PGs content in offspring were also observed, prenatal HMB supplementation upregulated leptin indirectly by enhancing the growth hormone IGF-1 and the pituitary-gonadal axis, which led to cell proliferation and more advanced collagen maturation. Those findings may explain the observed increase in tissue stiffness, which, among others, depends on both collagen content and the distribution of the chondrocytes within the tissue [61,62,63,64].

On the other hand, the chemical composition of AC could change during cartilage growth and maturation as a result of different joint loadings [25]. In our experiment, the HMB-supplemented male piglets had a higher body weight at weaning compared to the control groups (Table S1 in the Supplementary Materials). This feature could also be associated with higher joint loading or an adaptation to the amount of mechanical loading. Saadat et al. have reported an increase in PG content in a rabbit model due to physiologic in vivo cyclical joint loading localized in the DZ only, with unchanged collagen content [66]. This finding indicates a healthy joint response in which chondrocytes detect and react to the changes in their mechanical environment by activating proteoglycan biosynthesis.

However, being useful in the examination of biological samples, FTIR spectroscopic imaging has several limitations, including long image acquisition times and a vast amount of data received as a result of the analysis. Moreover, spectral analysis in some regions of FTIR spectra is complicated as features characteristic for lipids, proteins or carbohydrates often overlap each other [26,29,67]. In addition, spectroscopic data also contains plenty of unused information due to difficulty in identifying beneficial features. Therefore, additional analysis tools are needed to determine the differences in the distribution of cartilage chemical components after HMB supplementation. In view of these difficulties, principal component analysis (PCA) and artificial neural networks (ANNs) were applied. 

**Figure 4:**
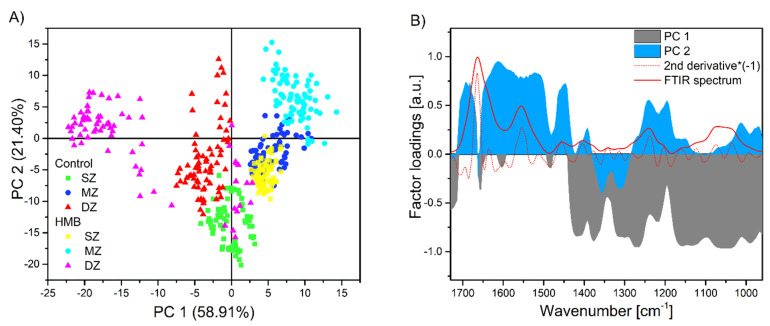
PCA score (**A**) and factor loading (**B**) plots of the first and the second principal components based on the infrared spectra of articular cartilages (AC) samples from the control and HMB-supplemented group in the region of 1730–960 cm^−1^. In (**B**), a solid red line corresponding to a typical FTIR spectrum and a dotted line to its second derivative (multiplied by −1) revealed the mid-infrared regions covered by factor loadings. HMB–supplemented group, SZ–superficial zone, MZ–middle zone, DZ–deep zone.

The principal component analysis is one of the most popular methods applied in the feature extraction process [68]. The PCA algorithm creates new features, lower in dimension, that project the original feature vectors into a new space. This reduction is achieved by a linear transformation to a new set of variables, which are uncorrelated and are ordered according to their importance in representing the original variables [68]. By numerous applications in engineering and biology [69,70,71], PCA proved to be useful and effective. In our present study, PCA supports resolving overlapping spectral features and provides further information about the main spectroscopic features and their variation for the set of AC samples. The results of spectral studies using PCA are shown in Figure 4A. The score plot displays several independent or overlapping clusters, using the two first components based on 75 spectra of the different AC samples. Figure 4A revealed distinct clustering of the control and HMB-supplemented groups in the SZ and DZ. There was a clear separation between the control and HMB groups in the SZ zone, with the DZ region overlapping with the control DZ and SZ. The DZ in the HMB-treated group was the most decentralized from other areas of the same AC sample. On the other hand, the PCA scores showed less separation between the control MZ and the SZ from the HMB-supplemented group. 

When the loading plots are observed (Figure 4B), the shape and position of PC 1 and PC 2 factor loadings are similar to a typical FTIR spectrum and its second derivative from the AC sample. Therefore, the first two components, PC 1 and PC 2, which can be easily interpreted concerning the biochemical components of the AC tissue, were considered for further discussion. The cumulative contribution of variance for the first two PCs was 80.31%, implicating that most of the information contained in the spectral matrix has been retained (Figure 4A). The PC 1 explains 58.91% of the total variance, and it is characterized by significant negative loadings (absolute value of the loading >0.7) of the aliphatic absorption bands (CH_3_, CH_2_, Table 1), the amide III and the PG-related bands. The PC 2 explains 21.40% of the total variance. Predominantly, the amide I and II absorption bands display large positive loadings (Figure 4B). Moreover, positive loadings at the amide III absorption region separated control from HMB-supplemented samples. The amide I and amide II regions are affected by the C=O, C–N and N–H vibrations characterized by collagen and PGs. Nevertheless, since approximately 60%–80% of the solid cartilage matrix is composed of collagen and PGs make the rest, the collagen molecules mainly dominate this region [72].

Since the PCA method is based on the linear relationships between variables, it was decided to apply an algorithm operating on nonlinear data processing, such as ANNs. Artificial neural networks are considered an excellent tool in classification and recognition problems. Unlike classical linear modelling, they facilitate solving complex nonlinear problems and simplify difficult multidimensional spaces by presenting the issue in the form of small-dimensional geometric relationships [73]. Moreover, ANNs can learn and are able to generalize, have fault tolerance and the possibility of parallel data processing [73,74]. In the presented research, two different ANNs were used: the multilayer perceptron network (MPL) and the Kohonen network. MLP is known for its outstanding results in classification problems mainly due to the fact that in the learning process, the activation level of the output neuron is transferred to the value of the output variable [74]. At the same time, the Kohonen network has two basic properties that distinguish it from other networks and make it a helpful tool for pattern detection: it is able to represent a multivariate input vector as a one- or two-dimensional output vector and can detect groups [75]. 

As presented in Table 3, 100% of the cases taking part in the MLP’s training and testing and 99% of the validation set were assigned correctly to the group. Furthermore, the activation levels of the output neurons ranged from 0.97 to 1.00 (Supplementary Material, Figure S5), which indicates that the network was entirely certain about the affiliation of analyzed cases to the given class.

**Figure 5:**
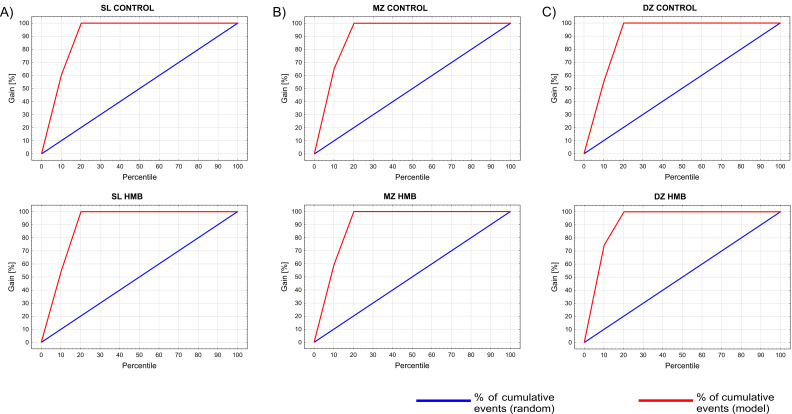
Gain charts constructed for the examined groups showing the effectiveness of a classification model calculated as the ratio between the results obtained with (red line) and without (blue line) the model. HMB—supplemented group, SZ—superficial zone, MZ—middle zone, DZ—deep zone.

Only one case from the validation set, which was derived from the HMB-supplemented DZ group, was assigned incorrectly to the control DZ group (confusion matrix available in the Supplementary Material, Table S2). Gain charts (Figure 5) constructed for examined groups show that by taking the top 20% (shown on the *x*-axis) of the cases classified into the corresponding category with the greatest certainty (maximum classification probability), we will correctly classify nearly 80% of all cases.

**Figure 6:**
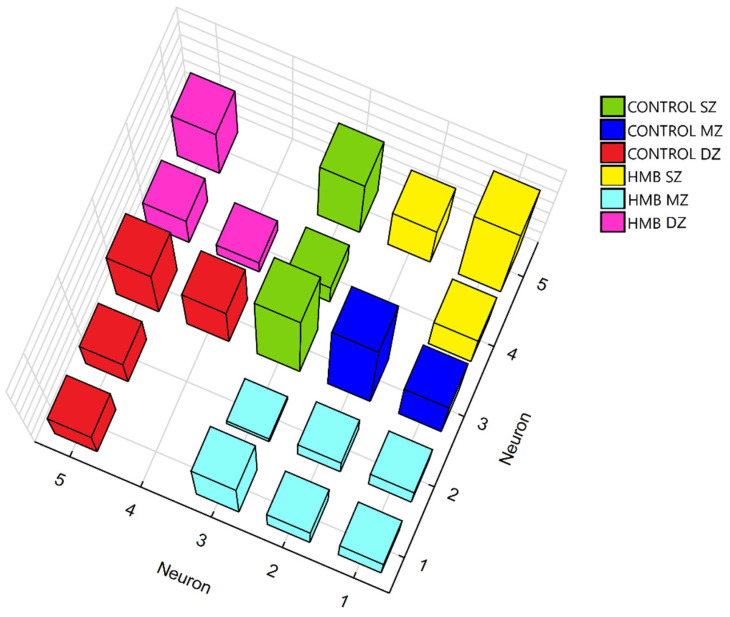
Clusters formed by neurons winning for specified cartilage zones in the control and HMB-supplemented group. HMB—supplemented group, SZ—superficial zone, MZ—middle zone, DZ—deep zone.

As a result of the training process (Table 4), clustering the SOM network with 25 neurons in the output zone divided the presented dataset into six groups (Figure 6). As can be seen, neurons winning in the training created clusters based on the similarity of given vectors describing the examined groups: control (SZ, MZ and DZ) and HMB (SZ, MZ and DZ). It might be observed that neurons winning for the middle zone and deep zone in the HMB group are located in the opposite corners of the square-shaped network, indicating that SOM considered them completely different. Similar behavior may be seen for the deep zone from the control group and the superficial zone from the HMB group. By contrast, groups such as the middle zone in the control and HMB groups are similar to a greater extent, so they are located close to each other on the SOM map.

**Figure 7:**
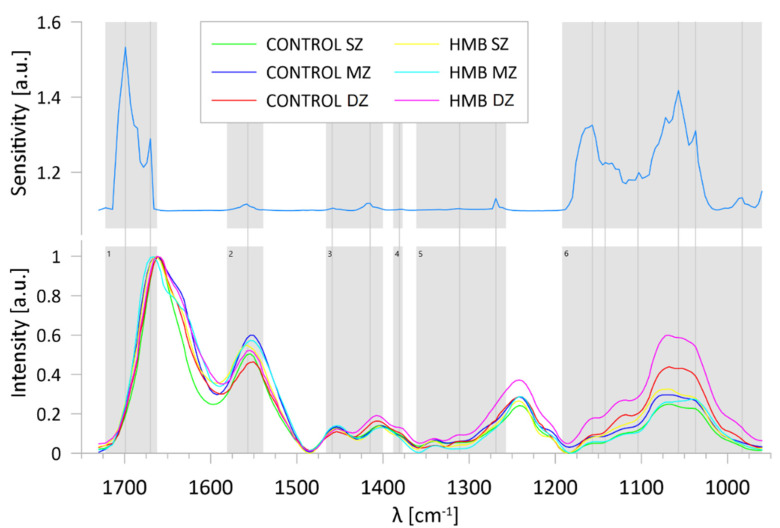
Wavenumber ranges indicated by the ANNs sensitivity analysis with marked peaks in each (grey, vertical lines) and corresponding spectra averaged for each group: (range 1) 1722-1667 cm^−1^; (range 2) 15981-1539 cm^−1^; (range 3) 1466-1400 cm^−1^; (range 4) 1385-1377 cm^−1^; (range 5) 1361-1257 cm^−1^; (range 6) 1192-960 cm^−1^; HMB—supplemented group, SZ—superficial zone, MZ—middle zone, DZ—deep zone.

A sensitivity analysis, performed to verify which wavenumbers contributed most to the classification (grouping) process, revealed that the ratios of the residuals’ sum of squares of the full model to its value when the examined variable was removed from the network were equal to or greater than one for 130 variables. The wavenumber ranges determined by the sensitivity analysis are presented in Figure 7. It shows that the most significant spectral regions for the correctness of the classification include ranges assigned to, among others, amide I (range 1), amide II (range 2) and amide III (range 5), glycosaminoglycans (range 4), CH_2_ and CH_3_ vibrations (range 3) and absorptions of carbohydrate moieties, including proteoglycans (range 6). Note that the spectra regions indicated in the sensitivity process are similar to those pointed in the analysis of PCA factor loadings, which leads to the conclusion that mentioned ranges are a kind of the fingerprint for the articular cartilage and allow identification of structural variations in the examined zones.

## 3. Materials and Methods

Articular cartilage samples were subjected to Fourier Transform Infrared microspectroscopy measurement. Spectra were collected from the three cartilage zones: superficial zone (SZ), middle zone (MZ) and deep zone (DZ). The obtained data were processed, and multidimensional analysis was applied to detect features that allow cartilage zones to be distinguished and alterations in their structure to be recognized. Additionally, AFM measurements were conducted to determine the structure and mechanical properties of the examined regions. The experiment was approved by The Local Ethics Committee for Animal Experimentation of University of Life Sciences in Lublin, Poland (reference number 2014/29) and was carried on following EU Directive 2010/63/EU.

### 3.1. Sample Preparation

Cartilage samples were harvested from 35-day old male piglets of Polish Large White (PLW) breed both from the control (C, *n* = 5) and experimental group (HMB, *n* = 5), which were fed a special diet during the fetal period. Pregnant sows received either a basal diet (control group) or the same diet supplemented with HMB at the dose of 0.2 g/kg of body weight/day (HMB group) from 70th until the 90th day of gestation. The gestation length did not differ between the control and HMB sows. All piglets were born by physiological partum, and no congenital changes were detected. The whole experimental set is described in detail in [18] and in [19]. Frozen tissue material was cut on an NX50 cryostat (Thermo Scientific, Waltham, MA, USA) into sections 7–10 µm thick (for FTIR) and 100 µm thick (for AFM). A total of 10 samples was obtained (*n* = 5 for the group) for each analysis (the scheme of sample distribution available in Supplementary Material, Figure S6). For FTIR measurements, the samples were mounted on low emissivity (low-e) microscope slides (Kevley Technologies, Chesterland, OH, USA) and subjected to analysis after air-drying for 1 h. In the case of AFM measurements, Superfrost Plus glass slides (Thermo Scientific, Braunschweig, Germany) covered with double-sided adhesive tape were used. Samples were stored at −20 °C until investigated.

### 3.2. Fourier Transform Infrared Microspectroscopy (FTIR Microspectroscopy) and Data Pre-Processing

FTIR microspectroscopy measurements were performed using a Hyperion 3000 Vis–IR microscope with a Vertex 70v spectrometer (Bruker Optik GmbH, Ettlingen, Germany) equipped with liquid nitrogen cooled with a 64 × 64 focal plane array (FPA) detector using a globar source. For each tissue sample (*n* = 10) seeded on the low-e slide, the IR mapping data were acquired in trans-reflectance mode using a 36× objective in the spectral range of 4000–750 cm^−1^ at 4 cm^−1^ spectral resolution. The samples were imaged to cover a 75 × 75 µm area, achieving a pixel resolution of 1.1 μm. Thus, for each pixel, the number of scans per spectrum was equal to 64.

The resulting FTIR spectra fixed in spectral imaging data were extracted and pre-processed using a CytoSpec™ version 2.00.01 software (Cytospec Inc., Boston, MA, USA). Before further analysis, the poor-quality spectra were removed from the dataset performing the quality test based on checking thickness, signal-to-noise ratio and enough high maximum absorbance in the region 1800–1600 cm^−1^. A noise-reduction algorithm, a second-order polynomial baseline correction and minimum–maximum normalization of all spectra were applied to optimize the spectral data from all tissue sections. The remaining spectra were cut to include only the fingerprint region between 1730 and 900 cm^−1^. The background spectra were collected from a pure low-e slide and subtracted automatically using the same acquisition procedure as tissue samples. For each experimental object (*n* = 10, 5 for the control and 5 for the experimental group) and from each cartilage zone, five regions of interest (ROI) with dimensions of 10 × 10 pixels, with 1 pixel corresponding to 1.19 × 1.19 µm^2^, were selected. The averaged FTIR spectra from those regions were subjected to further analysis to minimize the error resulting from variations in sample thickness. 

### 3.3. Chemical Mapping

Chemical maps were created by integrating the area under the curve for regions giving quantitative chemical information of the principal macromolecular components of the AC tissue. The integrated area of the amide I absorbance (1750–1590 cm^−1^) has been used to quantify the collagen content (CC), whereas the integrated area of the region that is assigned to the PG sugar ring (1140–965 cm^−1^) was correlated with the PGs content (PG) [23]. The ratio of the area of the CH_2_ side chain of collagen (1360–1325 cm^−1^) absorption to the amide II (1590–1490 cm^−1^) was used to determine collagen integrity (CI). All the parameters were collected within previously defined ROIs.

Statistical analysis of calculated parameters was performed using Statistica13.1 (TIBCO Software Inc. Palo Alto, CA, USA) and OriginPro 2016 (OriginLab Co., Northampton, MA, USA). The resulted dataset was checked for the distribution normality by the Shapiro–Wilk test, while the homogeneity of the variance was studied using the Levene test. Normally distributed variables were analyzed using a two-tailed Student’s *t*-test or *t*-test with Welch’s correction when data lacked equal variances; non-parametric data were analyzed using a Mann–Whitney U test. Data of collagen content, collagen integrity and proteoglycan content were analyzed using the GLM MIXED procedure of SAS (SAS Institute. Inc., Cary, NC, USA) with the dietary treatment as fixed effect and pig as a random effect. Analysis was performed separately for each histological layer of non-calcified articular cartilage to assess differences between the control (C) and supplemented (HMB) group in terms of the parameters characterizing the structure of AC within the studied zones. For all tests, a *p*-value <0.05 was established as statistically significant.

### 3.4. AFM Nanoindentation

AFM measurements were taken using a NanoWizard I AFM (JPK Instruments, Berlin, Germany) equipped with an inverse optical microscope (Axiovert 200, Carl Zeiss MicroImaging GmbH, Göttingen, Germany). For the imaging and indentation measurements, silicon nitride cantilevers (MLCT Microcantilever, Bruker, Mannheim, Germany) with a nominal spring constant of 0.1 N/m and pyramidal tips with the nominal radius of 20 nm were used. The actual values of the spring constant were determined using the thermal noise method [76,77] and averaged from the three trials. All the images (in height and vertical deflection domains) were taken with a slow scan rate of 1 Hz and with a resolution of 512 × 512 pixels per image. Additionally, 25 × 25 force-indentation curves were recorded for each sample. Measurements were taken for two samples from each examined group and were conducted in a phosphate-buffered saline (PBS) solution. Only extracellular matrix (ECM) was investigated within the analyzed zones, while chondrocytes with surrounding pericellular matrix (PCM) were skipped. Young’s modulus was calculated according to a modified Hertz model for a pyramidal indenter (Equation (1)) with the JPK Data Processing software (v.4.2.20, JPK Instruments AG, Berlin, Germany) application. The influence of the substrate was excluded by the reduction of the maximum indentation depth to a few per cent (lower than 10%) of the sample thickness (force-distant curves were analyzed up to 500 nm indentation depth).

(1) $$F=\frac{2tg\alpha E}{\pi \left(1-\nu^{2}\right)}\cdot \delta^{2},$$

where: *F*—force required to push the tip into the sample, *δ*—indentation depth, *α*—tip half-opening angle (17.5°), *ν*—the Poisson’s ratio (0.5 for biological materials [61,65,78,79]). 

Histograms of the stiffness distributions were calculated separately for each cartilage zone, the maxima of which were located with OriginPro 2016 (OriginLab Co., Northampton, MA, USA) by fitting Gaussian functions or, in the case of the deep zone, a linear combination of them [65]. Statistical differences between peaks’ values were checked by the *t*-Student test (Statistica13.1, TIBCO Software Inc. Palo Alto, CA, USA) after verifying that the test conditions were met.

### 3.5. Data Processing

IR spectra were randomly selected with the CytoSpec™ from the FTIR images of all ten articular cartilage samples. The 15 representative spectra were extracted from each sample and each region (*n* = 45 per section) within the previously defined ROI. Data were prepared in the form of the matrix X = [x_ij_]_d×m_, the elements of which represented the values of signal intensity (absorbance), lines (d) corresponded to subsequent observations and the variables (m) were the values of wave numbers expressed in cm^−1^. Since the sampling rate was equal to about 4 cm^−1^ (3.93 cm^−1^), 196 variables were obtained in the tested range. Consequently, the final spectral data set, containing 450 spectra in the spectral region of 1730–960 cm^−1^, was subjected to further analysis with PCA and ANNs application. 

### 3.6. Principal Component Analysis (PCA)

The principal component analysis (PCA) was performed (Statistica13.1, TIBCO Software Inc. Palo Alto, CA, USA) to reduce the number of variables (FTIR spectral vectors) into a few principal components (PCs). PC scores were achieved from data sets on correlation mode, and the variance was plotted on a 2D axis. The scree plots and the Kaiser–Guttmann criterion were used to select an optimum number of variables. The data matrix was scaled to the unitary standard deviation, which means that PCA was done on the correlation matrix, and all the variables were treated on an equal footing.

### 3.7. Artificial Neural Networks (ANNs)

The input for artificial neural networks was a set of four hundred and fifty 196-element vectors, the standardized components of which reflected the absorbance intensities depending on the value of the wavenumber. In addition, the data matrix was randomly divided into training, test and validation sets in the ratio of 75:15:15 and used as an input for classifying and grouping networks. 

MLP network, chosen to classify spectra into one of six groups, had one hidden layer with 18 neurons activated with logistic function and 6 outputs activated by the softmax function. The architecture of the classifying network was determined using the growth method. The MLP network was trained with the Broyden–Fletcher–Goldfarb–Shanno (BFGS) algorithm [73] for 100 epochs, with a constant learning coefficient of 0.01 and momentum of 0.80. The acceptance and rejection thresholds were 0.95 and 0.05, respectively, corresponding to the standard classification at the 95% confidence level. As an error function, cross-entropy (CE) was applied (Equation (2)) due to the fact that CE implementation allows interpreting output values as the probabilities of the object group membership [80].

(2) $$E_{CE}=-\sum_{i=1}^{N}y_{i}\left(\frac{d_{i}}{y_{i}}\right),$$

where: *y_i_*—real output value, *d_i_*—expected output value, *N*—the number of teaching pairs input-output.

Kohonen network (Self Organising Map, SOM), which aimed to group the spectra according to their similarity, was built with 25 output neurons. As an error function, quantization error (QE, Equation (3)) was used. QE measures the distance of the winning neuron weight vector from the input vector, i.e., the distance of the input pattern from the nearest center, which expresses the accuracy of mapping the input data by the winning neuron [75]. Since QE allows assessing the neuron map fitting to the input data, the network with the lowest error value was chosen.

(3) $$QE=\frac{1}{Q}\sum_{q=1}^{TQ}\|x-w\|,$$

where: ***x***—the input vector; ***w***—the weight vector of the winning neuron for the ***x*** vector.

SOM network was trained with 1000 epochs according to Kohonen WTM (winner takes most) rule (Equation (4)) [75], with decreasing learning rate (0.9–0.01) and neighborhood (from 5 to 0).

(4) $$w_{ij}\left(t+1\right)=w_{ij}\left(t\right)+\alpha \left(t\right)h_{ci}\left(t\right)\left[x_{j}-w_{ij}\left(t\right)\right],\;$$

where: *w_ij_(t+1)*—the element of the weight vector of the connection between the *i*-th neuron and the *j*-th input vector in the current learning cycle; *w_ij_(t)*—the element of the weight vector of the connection between the *i*-th neuron and the *j*-th input vector in the previous learning cycle; 0 < *α(t)* < 1—decreasing learning rate, *h_ci_(t)*—the neighborhood function decreasing in time with respect to the winning neuron, *t*—previous teaching cycle, *t+1*—current teaching cycle. 

For all the variables, sensitivity analysis was performed to determine the ranges that are crucial for classification. All the simulations were conducted in Statistica13.1 software (TIBCO Software Inc. Palo Alto, CA, USA).

## 4. Conclusions

To sum up, this is the first report combining FTIR imaging with multivariate analysis to study the effect of the HMB supplementation on the structure and properties of articular cartilage. Experimental results confirm that Fourier transform infrared microspectroscopy imaging has potential as a quantitative alternative to conventional methods such as histology and biochemical analysis to characterize the articular cartilage components. Moreover, it gives the possibility to obtain novel biochemical information from investigated samples in a non-destructive and no staining required fashion. Quantitative FTIR mapping has revealed both zonal- and experimental group-dependent changes within the AC tissue after the HMB treatment, which was reflected in mechanical features of cartilage proved by AFM measurements. The relatively higher amount of collagen content was localized in the superficial and the middle zone. At the same time, the distribution of proteoglycans significantly increased in the middle and deep zones compared to the untreated HMB group. These findings were consistent with our previous works utilizing traditional biochemical assays.

Furthermore, the application of the multivariate analysis to the spectral data revealed the existence of frequency ranges that allow for an unambiguous classification of the spectrum, which in the case of a heterogeneous tissue in which the boundary between individual zones is often challenging to define, may constitute the basis for creating a supporting method for this type of investigation. Finally, it should be underlined that only a few studies have applied machine learning techniques based on artificial neural networks for the analysis and processing of cartilage MIR spectral data [58]. Nevertheless, ANNs were considered a promising tool in this type of analysis. Moreover, since their potential implications for the cartilage condition diagnosis and monitoring were highlighted, it may imply direction for further research.

## Figures and Tables

**Table 1:** The most significant bands obtained from the second derivative FTIR spectra. An assignment of spectral features was collected according to the literature [23,25,26,29,57,59].

Wavenumber [cm^−1^]	Assignment of Second Derivative FTIR Bands of AC
1695, ~1660, 1635	80% ν(C=O), 20% ν(CN), τ(HOH), amide I from PGs, water
1550	60% τ(N–H), 30% ν(C–N), 10% ν(C–C), amide II
1457	δ_as_ (CH_3_)
1403	ν_s_(COO^−^) of GAGs
1378	δ_s_ (CH_3_) of GAGs
~1340	CH_2_ side-chain vibrations of collagen II
1238	ν_as_ SO_3_^−^ of sulphated GAGs with CH_2_ wagging vibration from the glycine backbone and proline side-chain
1204	ν(C−N) of amide III, δ(N−H) of collagen
1161	ν(C–O) of the carbohydrate residues
1122	ν_as_(C–O–S)
1080–1060	ν(C–O) of the carbohydrate residues in PGs, ν_s_SO_3_^−^ of sulphated GAGs
~1050	ν(C–O) of the carbohydrate residues in collagen and PGs
1033	ν(C–O) of the carbohydrate residues in collagen and PGs

The symbols concerning the vibrations assignment are related to the vibrational stretching mode (ν), deformational (δ), bending (τ), and symmetrical (s) and asymmetrical (as) modes.

**Table 2:** Values of Young’s modulus given as means ± SD. Statistically significant differences between groups (at *p* < 0.05) were signed by ^a^, ^b^, ^c^ and ^d^.

Group	Zone		Young’s Modulus [kPa]
Control	SZ		33.88 (±0.58) ^a^
HMB	SZ		88.82 (±1.18) ^b^
Control	MZ		29.16 (±0.67) ^a^
HMB	MZ		80.02 (±0.74) ^b^
Control	DZ	1st peak	40.02 (±1.03) ^a^
		2nd peak	77.58 (±0.79) ^b^
HMB	DZ	1st peak	42.72 (±1.51) ^c^
		2nd peak	81.38 (±1.19) ^d^

HMB—supplemented group, SZ—superficial zone, MZ—middle zone, DZ—deep zone, CC—collagen content, CI—collagen integrity, PG—proteoglycans content. The first peak in a bimodal distribution of Young’s modulus in the DZ is connected with the proteoglycan phase, while the second peak is attributed to the collagen phase [61,65].

**Table 3:** Results of the MLP network training process.

Network	Recognition Rate			Function		
	Training	Validation	Testing	Error	Activation (Hidden)	Activation (Output)
MLP BFGS 100	1.00	0.99	1.00	Entropy	Logistic	Softmax

**Table 4:** Results of the training process of SOM network with 25 output neurons.

Network	QE (Teaching)	QE (Test)	QE (Validation)	Teaching Algorithm (Epochs)
SOFM 196-25	0.86	1.02	0.93	Kohonen 1000

## Data Availability

Data reported in this manuscript will be available upon request.

## Supplementary Materials

The following are available online at https://www.mdpi.com/article/10.3390/ijms22179189/s1.
