# Supplementary material for: Fourier Transform Infrared Microspectroscopy Combined with Principal Component Analysis and Artificial Neural Networks for the Study of the Effect of β-Hydroxy-β-Methylbutyrate (HMB) Supplementation on Articular Cartilage
Source: Int J Mol Sci. 2021 Aug 25;22(17):9189. doi: 10.3390/ijms22179189 (PMC8430473; doi:10.3390/ijms22179189)
Supplement: Supplementary file 1 [file ijms-22-09189-s001.zip › Supplementary Material/SUPPLEMENTARY MATERIAL.pdf]

SUPPLEMENTARY MATERIAL

Fourier transform infrared microspectroscopy (FTIR-MS) combined with principal component analysis and artificial neural networks for the study of effect of β-hydroxy-β-methylbutyrate (HMB) supplementation on articular cartilage

Izabela Świetlicka [1*], Carina Prein [2,3], Hauke Clausen-Schaumann [2,4], Attila Aszodi [3], Marcin Arciszewski [5], Tomasz Blicharski [6], Mariusz Gagoś [7,8], Michał Świetlicki [9], Katarzyna Kras[5], Ewa Tomaszewska [10], Siemowit Muszyński [1], and Marta Arczewska [1*]

[1] Department of Biophysics, Faculty of Environmental Biology, University of Life Sciences in Lublin, 13 Akademicka St., 20-950 Lublin, Poland; izabela.swietlicka@up.lublin.pl (I.Ś.), marta.arczewska@up.lublin.pl (M.A.), siemowit.muszynski@up.lublin.pl (S.M)

[2] Center for Applied Tissue Engineering and Regenerative Medicine-CANTER, Munich University of Applied Sciences, Munich, Germany, carina.prein@uwo.ca (C.P), hauke.clausen-schaumann@hm.edu (H.C.-S.)

[3] Laboratory of Experimental Surgery and Regenerative Medicine (ExperiMed), Clinic for General, Trauma and Reconstructive Surgery, University of Munich, 82152 Planegg, Germany, carina.prein@uwo.ca (C.P.), attila.aszodi@med.uni-muenchen.de (A.A.)

[4] Center for Nanoscience-CeNS, Munich, Germany, hauke.clausen-schaumann@hm.edu (H.C.-S.)

[5] Department of Animal Anatomy and Histology, University of Life Sciences in Lublin, 20-950 Lublin, Poland; marcin.arciszewski@up.lublin.pl (M.A.), katarzyna.kras@up.lublin.pl (K.K.)

[6] Chair and Department of Rehabilitation and Orthopedics, Medical University in Lublin, 8 Jaczewskiego St., 20-090 Lublin, Poland; tomasz.blicharski@umlub.pl (T.B.)

[7] Department of Cell Biology, Maria Curie Sklodowska University, Akademicka 19, 20-031 Lublin, Poland; mariusz.gagos@poczta.umcs.lublin.pl (M.G.)

[8] Department of Biochemistry and Molecular Biology, Faculty of Medicine, Medical University of Lublin, Chodzki 1, 20-093 Lublin, Poland; mariusz.gagos@poczta.umcs.lublin.pl (M.G.)

[9] Department of Applied Physics, Faculty of Mechanical Engineering, Lublin University of Technology, 20-618 Lublin, Poland; m.swietlicki@pollub.pl (M.Ś.)

[10] Department of Animal Physiology, Faculty of Veterinary Medicine, University of Life Sciences in Lublin, 12 Akademicka St., 20-950 Lublin, Poland; ewaRST@interia.pl (E.T.)

* Author to whom correspondence should be addressed.: izabela.swietlicka@up.lublin.pl (I.Ś.), marta.arczewska@up.lublin.pl (M.A.)

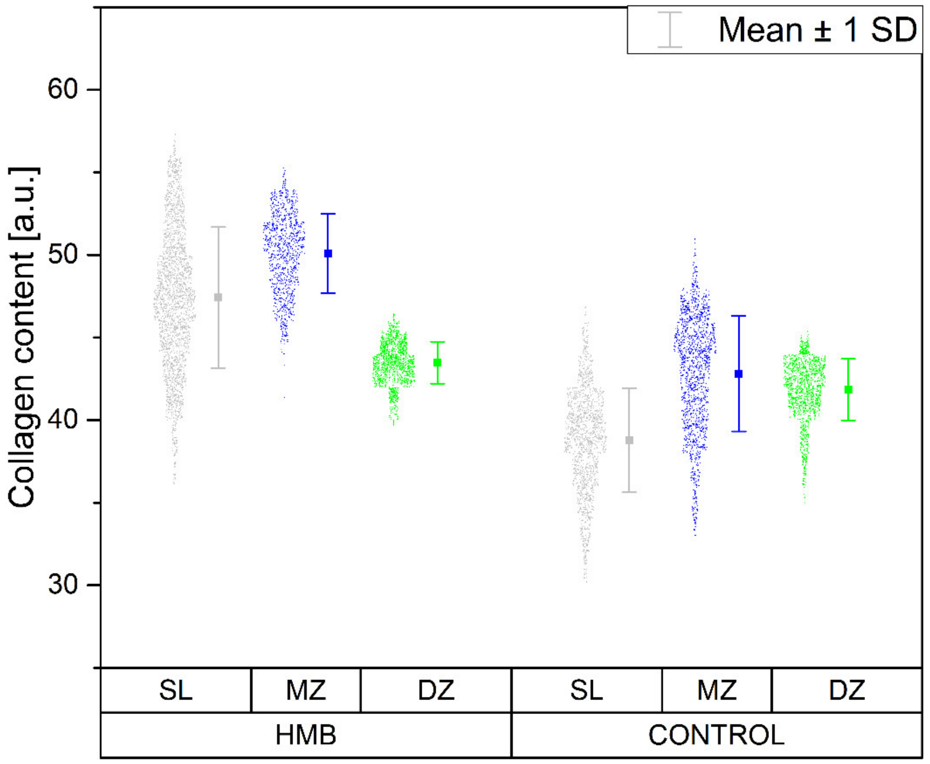

Figure S1. Scatter of collagen content (CC) values according to the cartilage zone and examined group. HMB – supplemented group, SL – superficial layer, MZ – middle zone, DZ – deep zone, CC – collagen content, CI – collagen integrity, PG – proteoglycans content

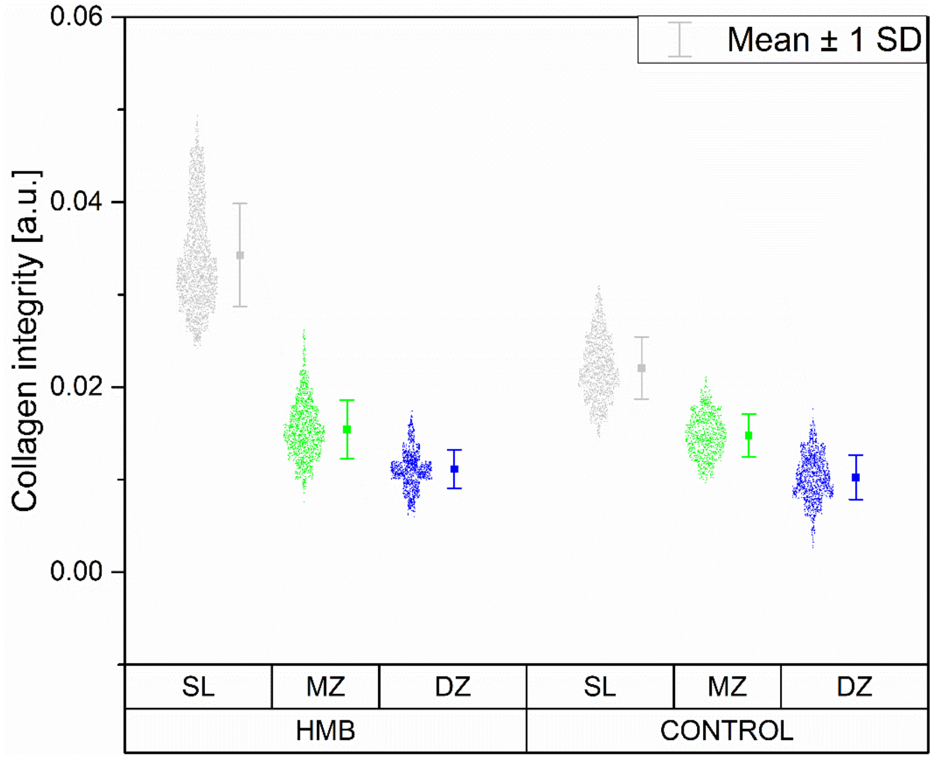

Figure S2. Scatter of collagen integrity (CI) values according to the cartilage zone and examined group. HMB – supplemented group, SL – superficial layer, MZ – middle zone, DZ – deep zone, CC – collagen content, CI – collagen integrity, PG – proteoglycans content

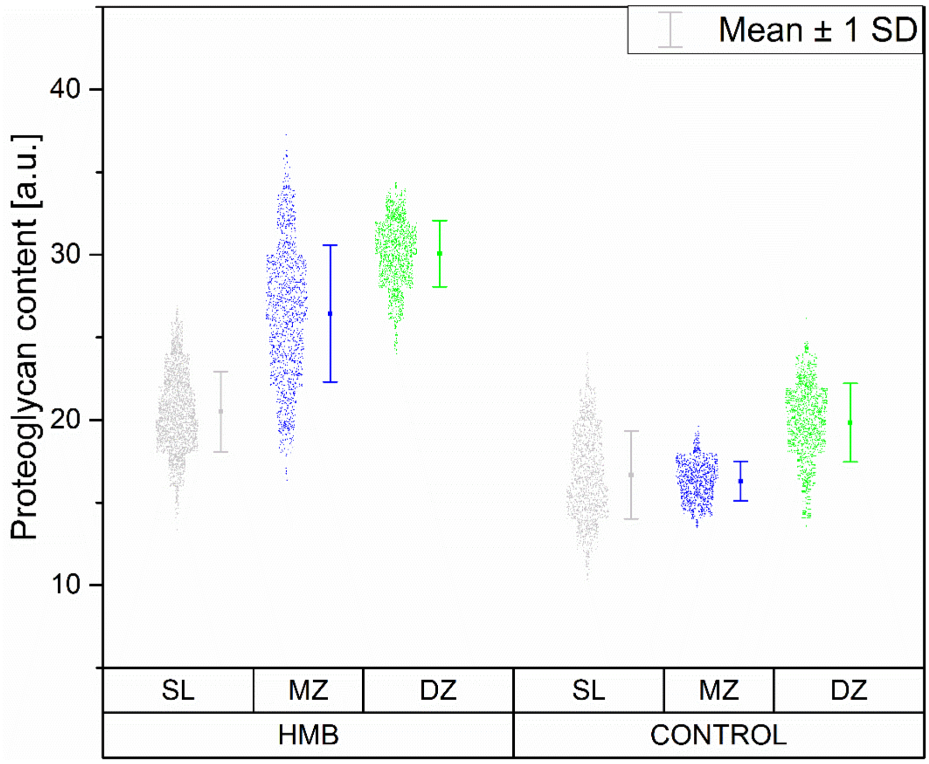

Figure S3. Scatter of proteoglycans content (PG) values according to the cartilage zone and examined group. HMB – supplemented group, SL – superficial layer, MZ – middle zone, DZ – deep zone, CC – collagen content, CI – collagen integrity, PG – proteoglycans content

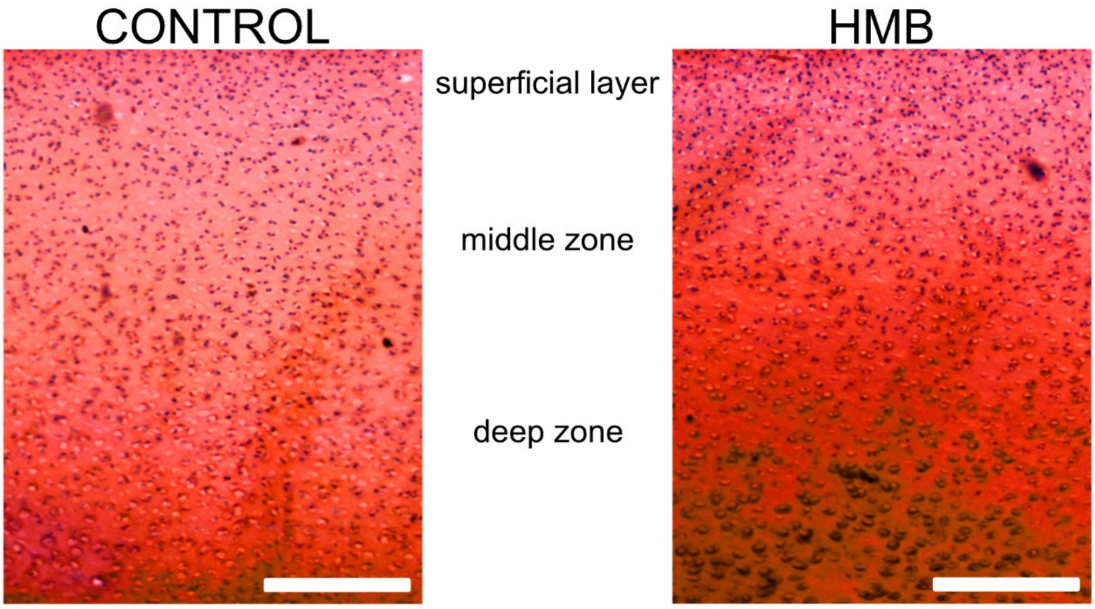

Figure S4. Representative images of safranin O staining of articular cartilage from the femur of 35-day old male piglets from control and HMB groups. More uniform and lower proteoglycan content (displaying weaker staining) can be observed in the articular cartilage from the Control group, while the HMB group demonstrated moderate and layer-dependent staining linked with higher content of proteoglycans. All the scale bars represent 100 μm.

Table S1. The effect of maternal HMB treatment on body weight in weaned piglets.

Treatment	Body weight [g]	Bone weight [g]
Main effect supplementation		
Control	6635	30.2
HMB	8163	42.5
Main effect sex		
Control male	6775	31.4
HMB male	8507	43.5
Control female	6495	29.1
HMB female	7820	41.4
Pooled SEM	212	1.6
Main effects and interactions		
Supplementation	<0.001	<0.001
Sex	0.192	0.243
Supplementation × sex	0.268	0.880
Weight at birth[a]	0.439	0.886

Data given are mean (n = 6); SEM: standard error of the means; [a]Weight at birth was used as covariate. With the permission from [1] (cited as 19 in the main article).

Table S2. Confusion matrix for the MLP classifying network.

Actual / Predicted	DZ CONTROL	DZ HMB	MZ CONTROL	MZ HMB	SL CONTROL	SL HMB
**TEACHING**						
**DZ CONTROL**	**58**	0	0	0	0	0
**DZ HMB**	0	**43**	0	0	0	0
**MZ CONTROL**	0	0	**49**	0	0	0
**MZ HMB**	0	0	0	**54**	0	0
**SL CONTROL**	0	0	0	0	**53**	0
**SL HMB**	0	0	0	0	0	**59**
**VALIDATION**						
**DZ CONTROL**	**9**	1	0	0	0	0
**DZ HMB**	0	**17**	0	0	0	0
**MZ CONTROL**	0	0	**13**	0	0	0
**MZ HMB**	0	0	0	**15**	0	0
**SL CONTROL**	0	0	0	0	**6**	0
**SL HMB**	0	0	0	0	0	**6**
**TEST**						
**DZ CONTROL**	**8**	0	0	0	0	0
**DZ HMB**	0	**14**	0	0	0	0
**MZ CONTROL**	0	0	**13**	0	0	0
**MZ HMB**	0	0	0	**6**	0	0
**SL CONTROL**	0	0	0	0	**16**	0
**SL HMB**	0	0	0	0	0	**10**

HMB – supplemented group, SL – superficial layer, MZ – middle zone, DZ – deep zone, CC – collagen content, CI – collagen integrity, PG – proteoglycans content

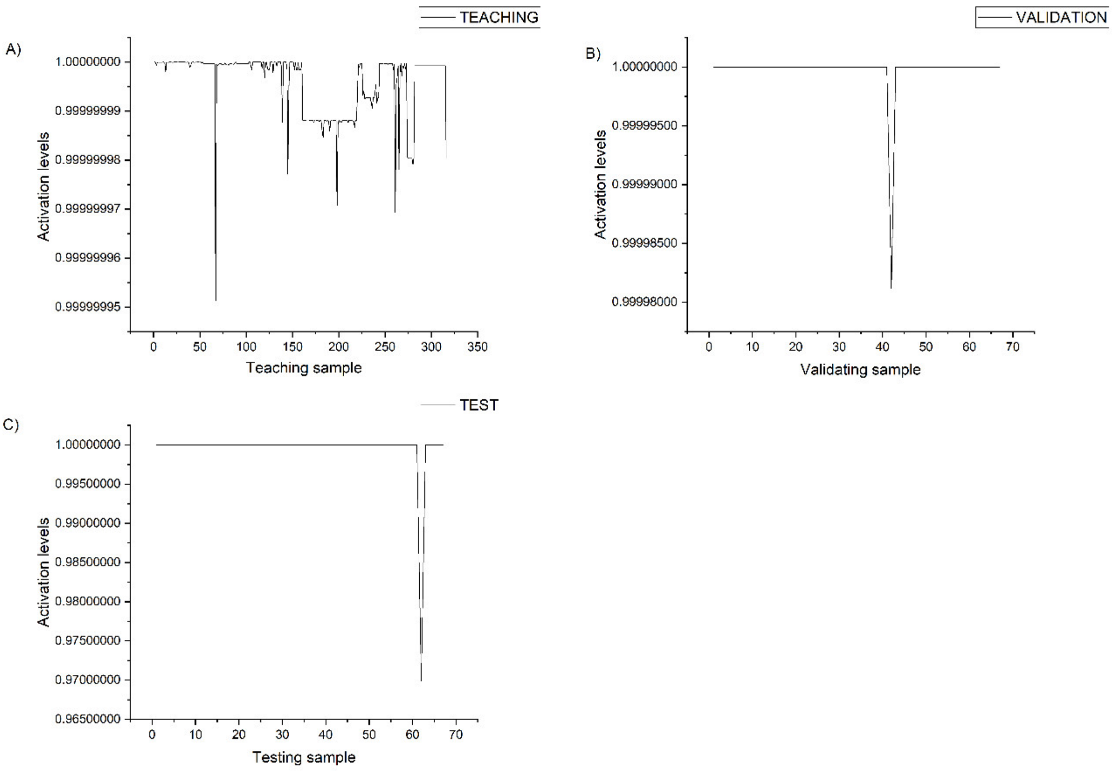

Figure S5. Activation levels of MLP network for teaching, validation and testing set.

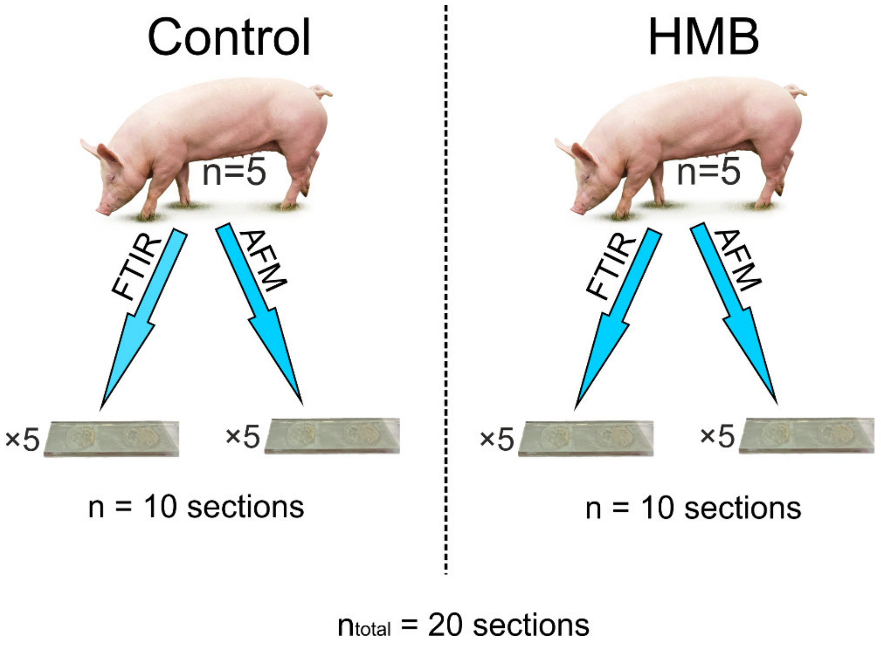

Figure S6. Sample distribution according to the analysis.

1. Tomaszewska, E.; Muszyński, S.; Dobrowolski, P.; Wiącek, D.; Tomczyk-Warunek, A.; Świetlicka, I.; Pierzynowski, S.G. Maternal HMB treatment affects bone and hyaline cartilage development in their weaned piglets via the leptin/osteoprotegerin system. *Journal of Animal Physiology and Animal Nutrition* **2019**, *103*, 626-643, doi:https://doi.org/10.1111/jpn.13060.
