# Supplementary figures and images for: Fourier Transform Infrared Microspectroscopy Combined with Principal Component Analysis and Artificial Neural Networks for the Study of the Effect of β-Hydroxy-β-Methylbutyrate (HMB) Supplementation on Articular Cartilage
Source: Int J Mol Sci. 2021 Aug 25;22(17):9189. doi: 10.3390/ijms22179189 (PMC8430473; doi:10.3390/ijms22179189)

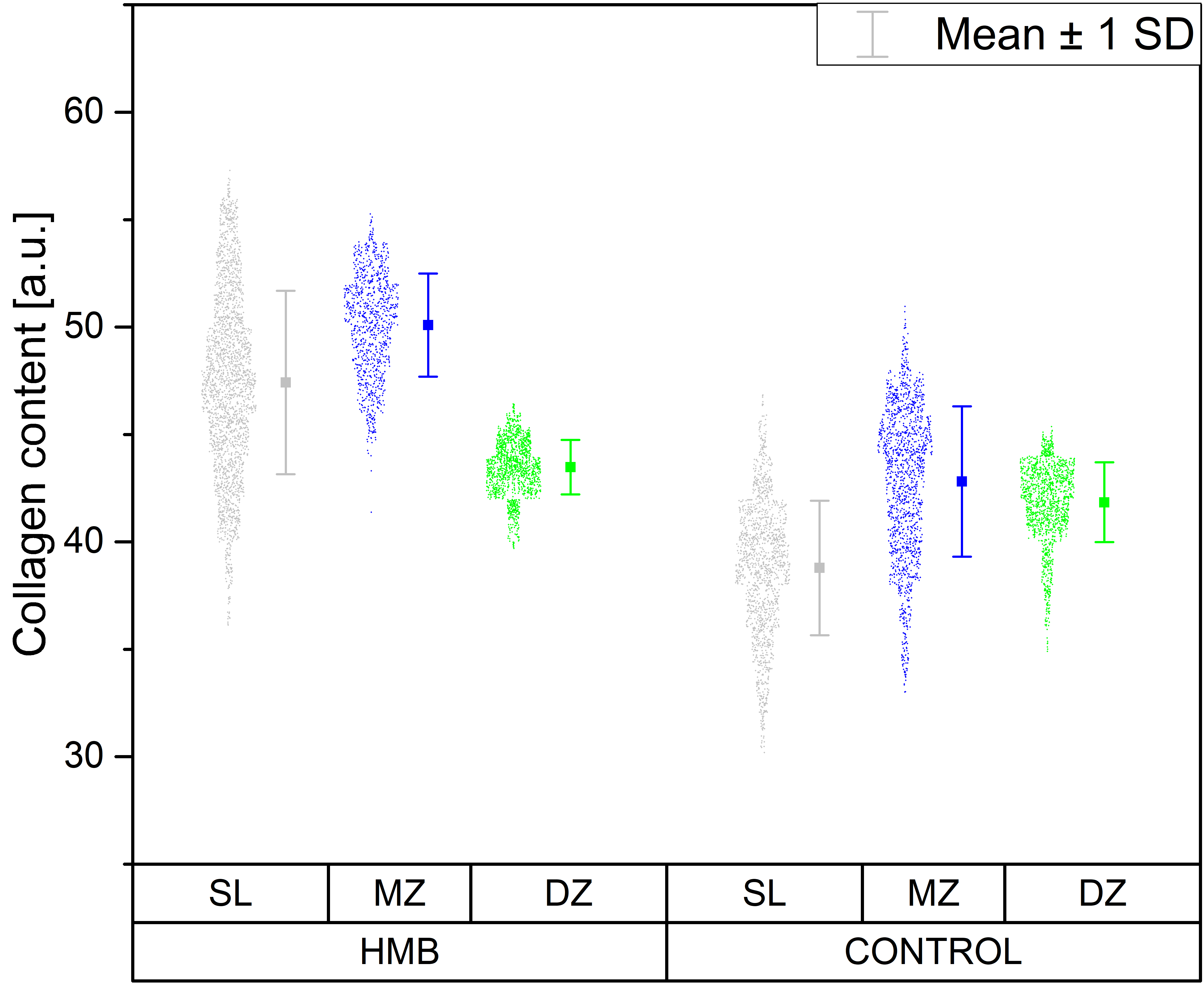

Supplement: Supplementary file 1 [file ijms-22-09189-s001.zip › Supplementary Material/Figure S1.tif]

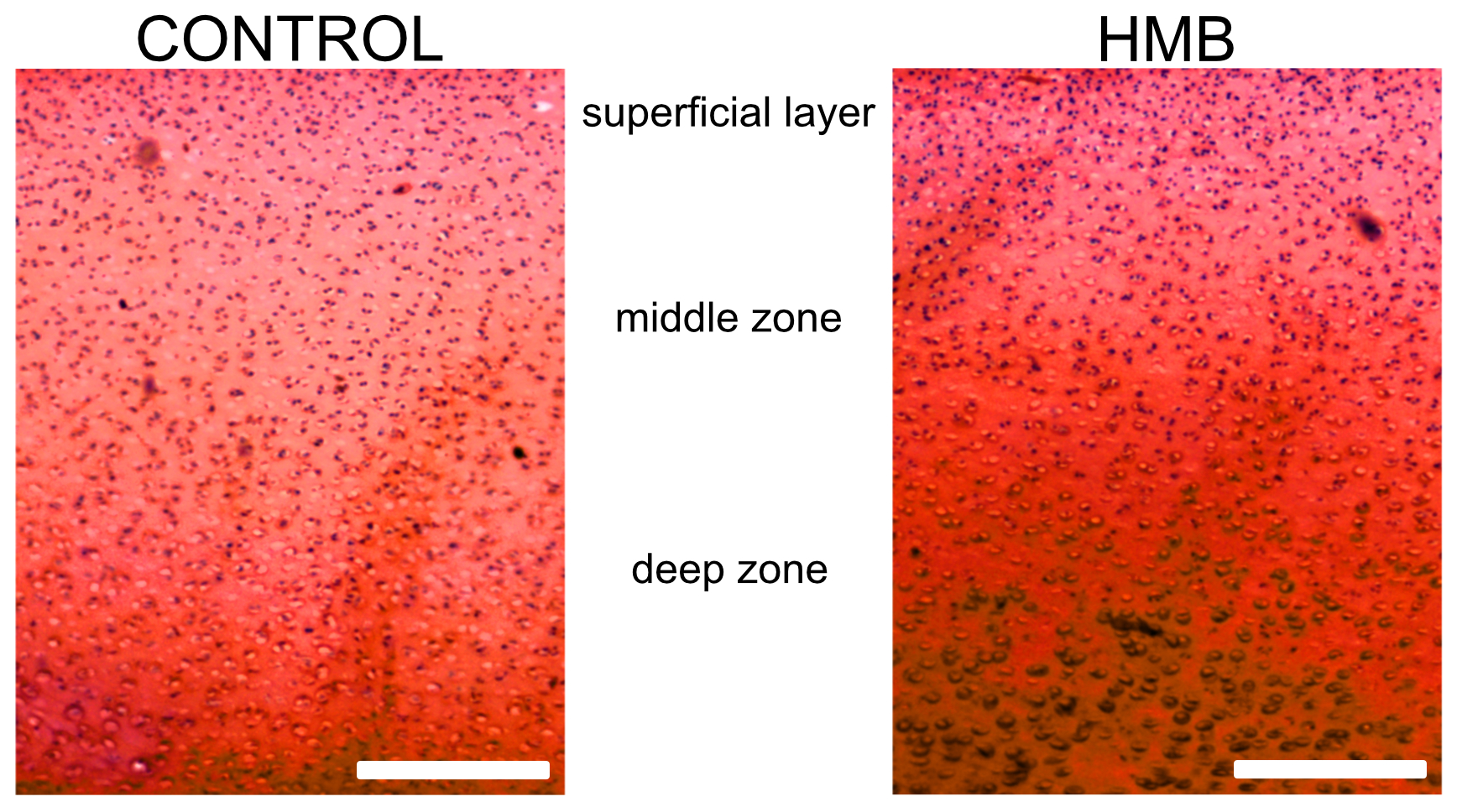

Supplement: Supplementary file 1 [file ijms-22-09189-s001.zip › Supplementary Material/Figure S4.jpg]

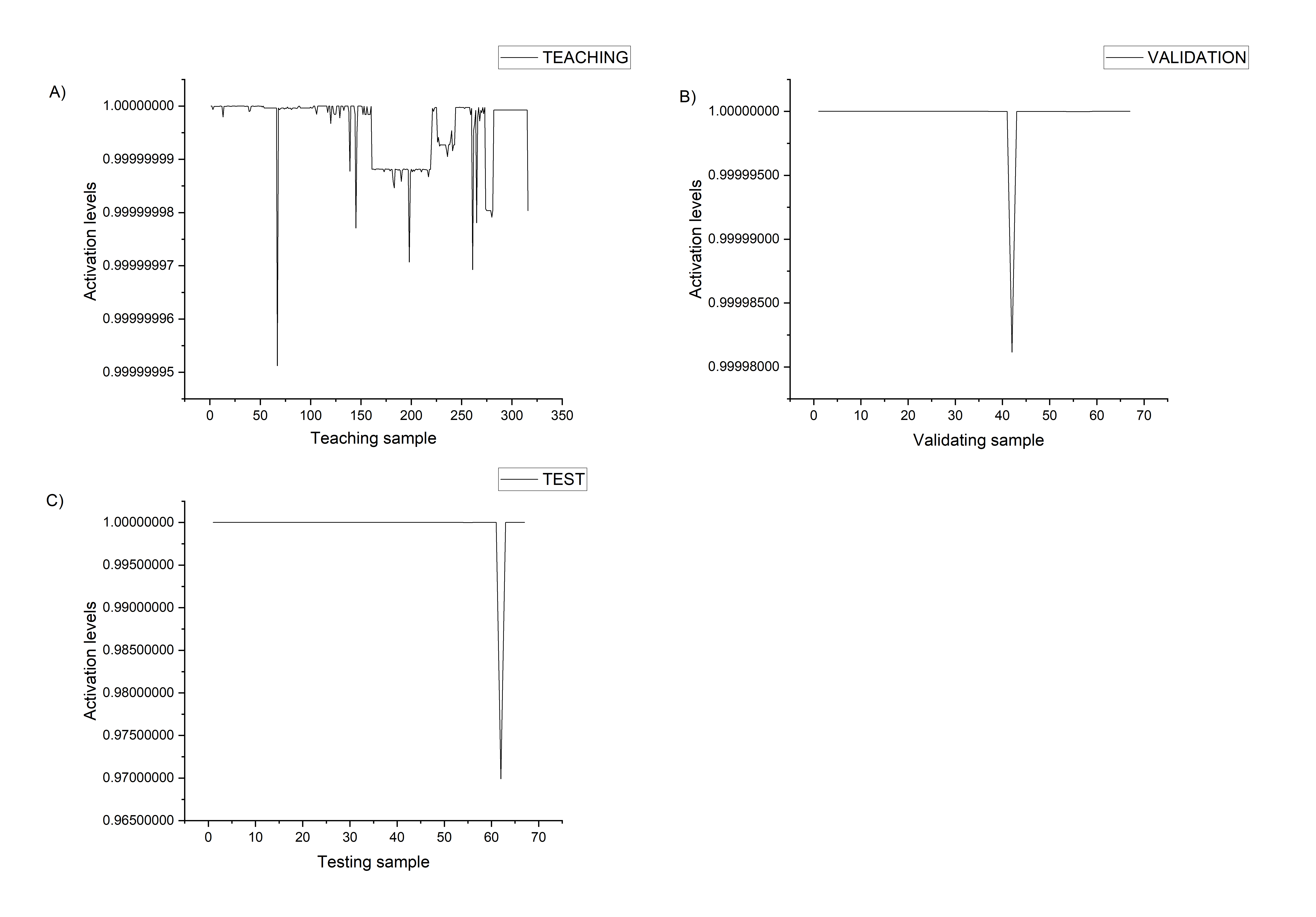

Supplement: Supplementary file 1 [file ijms-22-09189-s001.zip › Supplementary Material/Figure S5.tif]

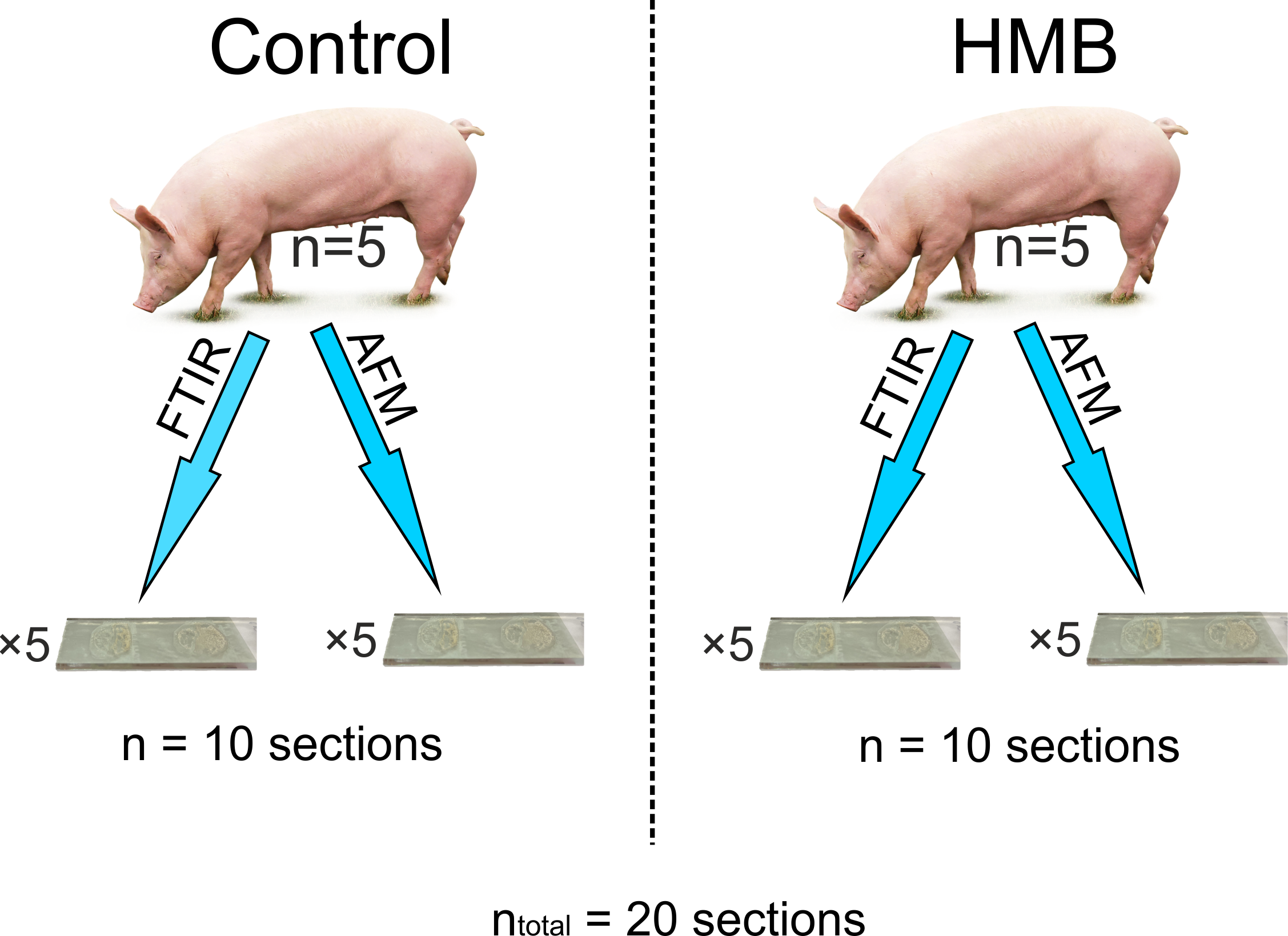

Supplement: Supplementary file 1 [file ijms-22-09189-s001.zip › Supplementary Material/Figure S6.tif]
